# Impact of global work index on obesity paradox in heart failure with advanced left ventricle remodeling

**DOI:** 10.3389/fcvm.2026.1693862

**Published:** 2026-02-17

**Authors:** Roubai Pan, Xierenayi Tudi, Weiwei Jiang, Xiao Zong, Yi Ni, Rong Tao, Qin Fan

**Affiliations:** Department of Cardiovascular Medicine, Ruijin Hospital, Shanghai Jiao Tong University School of Medicine, Shanghai, China

**Keywords:** body mass index, global work index, heart failure, left ventricular mass index, myocardial work

## Abstract

**Aims:**

This study aimed to examine the role of myocardial work in the obesity paradox among heart patients with heart failure and advanced left ventricular remodeling.

**Methods:**

In this *post hoc* analysis, 515 consecutive patients with LVEF ≤ 50% who were hospitalized between May 2016 and December 2020 were included and followed for a median of 37.4 months. Left ventricular mass index (LVMI) was used to define advanced left ventricular remodeling. The primary endpoint was major adverse cardiovascular events (MACE). We assessed the prognostic impact of obesity at different levels of the global work index (GWI) in patients with advanced left ventricular remodeling.

**Results:**

Of the 515 patients, 330 were classified as having advanced left ventricular remodeling based on LVMI thresholds (men ≥110 g/m^2^, women ≥120 g/m^2^). In this subgroup, a BMI ≥ 28 kg/m^2^ was associated with a lower risk of MACE. These 330 patients were further stratified into higher GWI (*n* = 139) and lower GWI (*n* = 191) subgroups. In the higher GWI subgroup, both BMI ≥ 28 kg/m^2^ and continuous BMI values were associated with a reduced risk of MACE. No such association was observed in the lower GWI subgroup.

**Conclusion:**

Myocardial work index modifies the relationship between BMI and prognosis in heart failure. GWI should be considered when evaluating the obesity paradox in patients with left ventricular systolic dysfunction, particularly those with advanced left ventricular remodeling.

## Graphical Abstract

**Figure:**
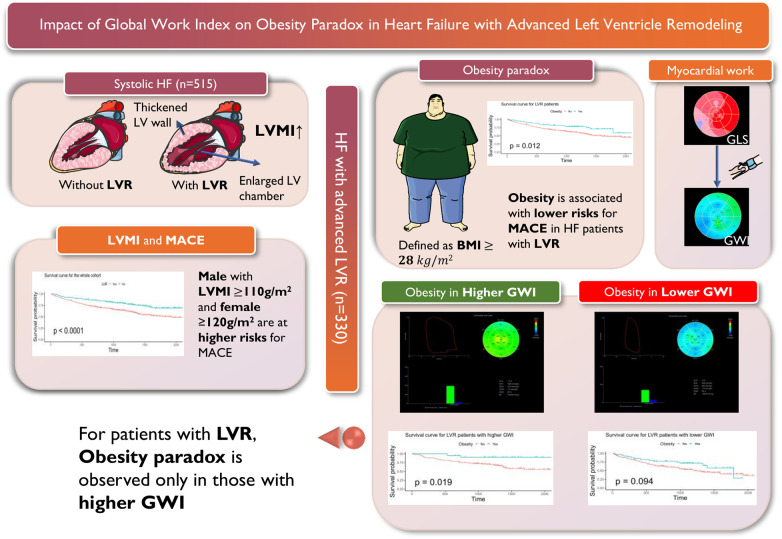
This figure illustrates the relationship between obesity, myocardial work, and major adverse cardiovascular events (MACE) in patients with systolic heart failure (HF) and advanced left ventricular remodeling (LVR). The left panel shows the comparison between HF patients with and without LVR. We use left ventricular mass index (LVMI) to quantitively depict LVR, as it is associated with LV wall thickening and LV chamber enlargement. The Kaplan-Meier analysis shows that male with LVMI ≥ 110 g/m^2^ and female with LVMI ≥ 120 g/m^2^ are at higher risks for MACE. The upper middle panel presents the survival analysis for patients with HF and advanced LVR, highlighting the association of obesity (defined as BMI ≥ 28 kg/m^2^) with lower risks for MACE. The upper right panel shows the assessment of global work index (GWI), by incorporating global longitudinal strain (GLS) and non-invasive systolic blood pressure. The bottom right panel distinguishes between obesity in higher and lower levels of GWI (grouped according to whether GWI is greater than 900 mmHg), demonstrating that the obesity paradox is only observed in patients with higher GWI, as indicated by the survival curves for patients with higher GWI (p = 0.019) and lower GWI (p = 0.094).

## 1 Introduction

Obesity is a major risk factor for cardiovascular disease and adversely affects both left ventricular (LV) structure and function (1), increasing the risk of incident heart failure (HF). Paradoxically, however, obesity appears to be associated with improved survival in patients with established HF—a phenomenon termed the “obesity paradox” (2).

Several hypotheses may explain this paradox. Obese patients may possess greater metabolic reserves, be less susceptible to cardiac cachexia (3), and often present with more symptoms, leading to earlier diagnosis and treatment (4). They may tolerate higher doses of cardioprotective medications. Epidemiological data have shown a U-shaped or J-shaped relationship between body mass index (BMI) and prognosis in HF, with the most favorable hazard ratio (HR) observed at a BMI of 32–33 kg/m^2^ (5). Patients with class I obesity (BMI 30–34.9 kg/m^2^) typically experience better outcomes than those in other BMI categories (6).

Given the association between obesity, LV structure, and HF prognosis, we investigated whether obesity retains prognostic significance in patients with advanced left ventricular remodeling (LVR). LVR is characterized by pathological cardiomyocyte hypertrophy and eccentric LV enlargement. The left ventricular mass index (LVMI)—derived from LV dimensions and wall thickness—is a key echocardiographic measure of LV geometry and a recognized predictor of adverse outcomes in HF (7). In this study, elevated LVMI was used to identify patients with advanced LVR.

Myocardial workload represents a major stimulus for LV remodeling; pressure overload promotes cardiac fibrosis and ultimately leads to HF. Myocardial work has emerged as a sensitive marker for the early HF diagnosis and prognostic assessment of heart failure (8, 9). Pressure–volume loop analysis derived from speckle-tracking echocardiography enables non-invasive measurement of myocardial work (10). Global longitudinal strain (GLS) is among the most well-known myocardial work parameters for evaluating LV performance. Recent studies suggest that the global work index (GWI) outperforms GLS in identifying coronary heart disease and other heart conditions associated with high afterload. Among myocardial work indices, GWI reflects the total work performed by the heart during systole. Moreover, GWI has been reported to remain stable across different types of LVR, such as eccentric or concentric remodeling (11). Thus, GWI holds considerable potential for assessing LV capacity and risk stratification in patients with HF and advanced LVR.

Since obesity is closely associated with cardiac workload, we aimed to evaluate whether the prognostic effect of obesity varies according to myocardial work capacity, as measured by GWI, in patients with advanced LVR.

## 2 Methods

### 2.1 Study design and patients

**Figure 1:**
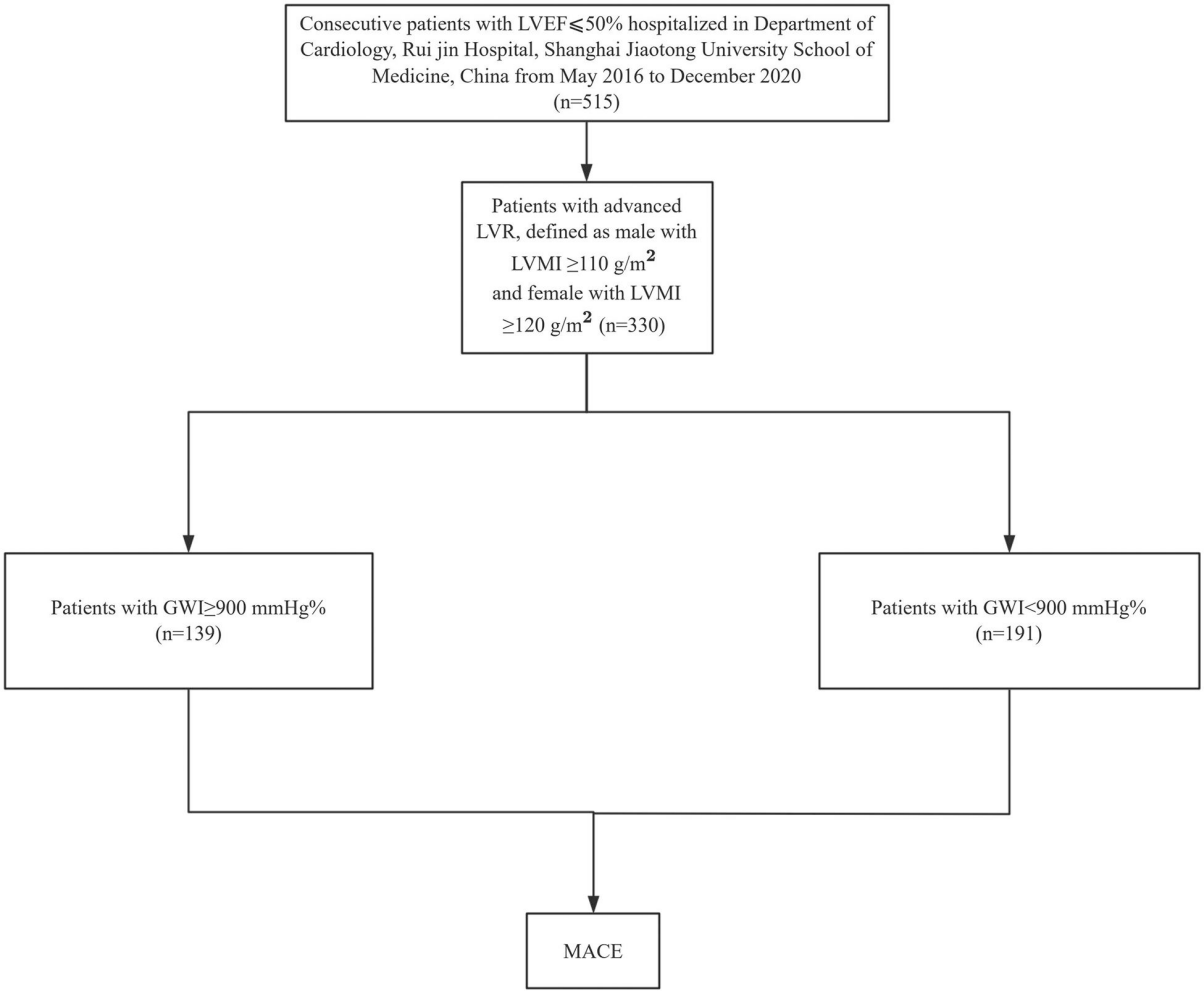
Flowchart for patient selection and study design.

This study was a *post hoc* analysis including 515 consecutive patients with HF of Chinese descent hospitalized at the Department of Cardiology, Ruijin Hospital, affiliated with Shanghai Jiao Tong University School of Medicine, between May 26, 2016 and December 4, 2020. The patient selection process and study design are summarized in Figure 1. The inclusion criteria were as follows: (1) presence of underlying heart disease that could lead to HF; and (2) LV systolic dysfunction, defined as an LV ejection fraction (LVEF) ≤ 50%. Demographic information, baseline measurements, and laboratory test data were retrospectively collected from medical records during hospitalization. Medication doses for all patients were adjusted according to blood pressure, heart rate, symptoms, and laboratory findings. Final dose adjustments were made within 2 days before discharge, and body weight was assessed after medication doses had been stabilized. Patients were scheduled for outpatient follow-up visits every 1–2 months during the first 6 months after discharge. For patients receiving optimized HF therapy, follow-up intervals were extended to at least every 3 months.

The study was conducted in accordance with the ethical guidelines of the 1975 Declaration of Helsinki and was approved by the institutional review committee of Ruijin Hospital, Shanghai Jiao Tong University School of Medicine. Written informed consent for data collection was obtained from all participants prior to screening.

### 2.2 Echocardiographic quantification

Baseline two-dimensional and Doppler transthoracic echocardiography was performed using a Vivid E9 imaging system with an M5S transducer (General Electric, Milwaukee, WI, USA) by experienced cardiac sonographers. All echocardiographic studies were reviewed by board-certified cardiologists. LVEF was calculated using the Simpson biplane method. LV end-diastolic diameter (LVEDD), interventricular septum, and posterior wall thickness were measured at end-diastole in the parasternal long-axis view. These measurements were used to calculate the LV mass (LVM) using the following formula: $\mathrm{LVM}(g)=0.8\;\times \;1.04\;\times \;({\left(\mathrm{IVS}+\;\mathrm{LVEDD}+\mathrm{PWT}\right)}^{3}-\mathrm{LVED}D^{3}\}+0.6$. LVM was then indexed to body surface area to obtain the LVMI, in accordance with the American Society of Echocardiography/European Association of Cardiovascular Imaging guidelines (12).

Two-dimensional speckle-tracking analysis was performed using EchoPAC Software, version 202 (General Electric). Valve event timings were assessed using pulsed-wave Doppler. Mitral valve opening and closure times were measured in the apical four-chamber view at the level of the mitral valve leaflets, whereas aortic valve opening and closure were assessed in the apical long-axis view at the LV outflow tract. Two-dimensional images were acquired at frame rates of 40–80 frames/s from the apical long-axis, four-chamber, and two-chamber views for the calculation of GLS. Blood pressure was measured immediately after echocardiography in the left lateral decubitus position using a sphygmomanometer. Peak LV systolic pressure was assumed to be equal to the peak cuff pressure. A non-invasive LV pressure curve was constructed by synchronizing valve events with cuff pressure. Integration of LV strain and pressure data yielded segmental and global myocardial work indices (10). GWI was calculated as the average of segmental values from mitral valve closure to mitral valve opening. The following additional parameters were calculated: global constructive work (GCW), global waste work (GWW), and global work efficiency (GWE), with GWE calculated as follows: GWE=GCW/(GCW+GWW)×100%**.**

### 2.3 Outcomes and follow-up

The primary endpoint was major adverse cardiovascular events (MACE), defined as cardiovascular mortality or hospitalization for HF. Cardiovascular death was defined as death due to HF decompensation, acute HF, myocardial infarction, fatal arrhythmia, or sudden cardiac death. The diagnosis of HF was based on clinical symptoms, laboratory tests, and radiologic evidence. Outcome data were collected by reviewing medical records and/or conducting telephone follow-up and were subsequently verified by an independent group of clinicians.

### 2.4 Statistical analysis

Statistical analysis was performed using R software, version 4.2.2. Continuous variables are presented as mean ± standard deviation (SD), and categorical variables are presented as counts and proportions. Patients were dichotomized based on LVMI using restricted cubic spline analysis and further categorized according to GWI levels, also derived from the same method. Obesity was defined as BMI ≥ 28 kg/m^²^. Baseline characteristics between subgroups were compared using Student's *t*-test for continuous variables and Pearson's chi-square test for categorical variables. The correlation between two continuous variables was assessed using Pearson’s correlation analysis. Time-to-event data were analyzed using Kaplan–Meier curves, and differences between subgroups were compared using the log-rank test. Cox proportional hazards regression was used to assess the prognostic value of continuous and dichotomous BMI, LVMI, and GWI. Variables such as sex, age, hypertension, diabetes mellitus, ischemic etiology, estimated glomerular filtration rate (eGFR), renin–angiotensin–aldosterone system, beta-blocker and mineralocorticoid receptor antagonist medication use, and N-terminal pro-brain natriuretic peptide (NT-proBNP) were adjusted for in multivariate Cox analysis. Restricted cubic spline analysis with knots selected by the Akaike information criterion was performed to evaluate the prognostic value of LVMI, GWI, and BMI. The median value of each variable in the study cohort served as the reference point in the spline models. The statistical significance of the identified cutoff values was further verified using Kaplan–Meier analysis and Cox regression. A two-sided *p*-value ≤ 0.05 was considered statistically significant for all tests.

## 3 Results

### 3.1 Patient characteristics and follow-up

Patients were followed for a mean duration of 37.4 ± 19.8 months. During the follow-up period, 183 of the 515 patients experienced a MACE, including 55 cardiovascular deaths and 128 hospitalizations for HF.

### 3.2 Predictive value of LVMI

**Figure 2:**
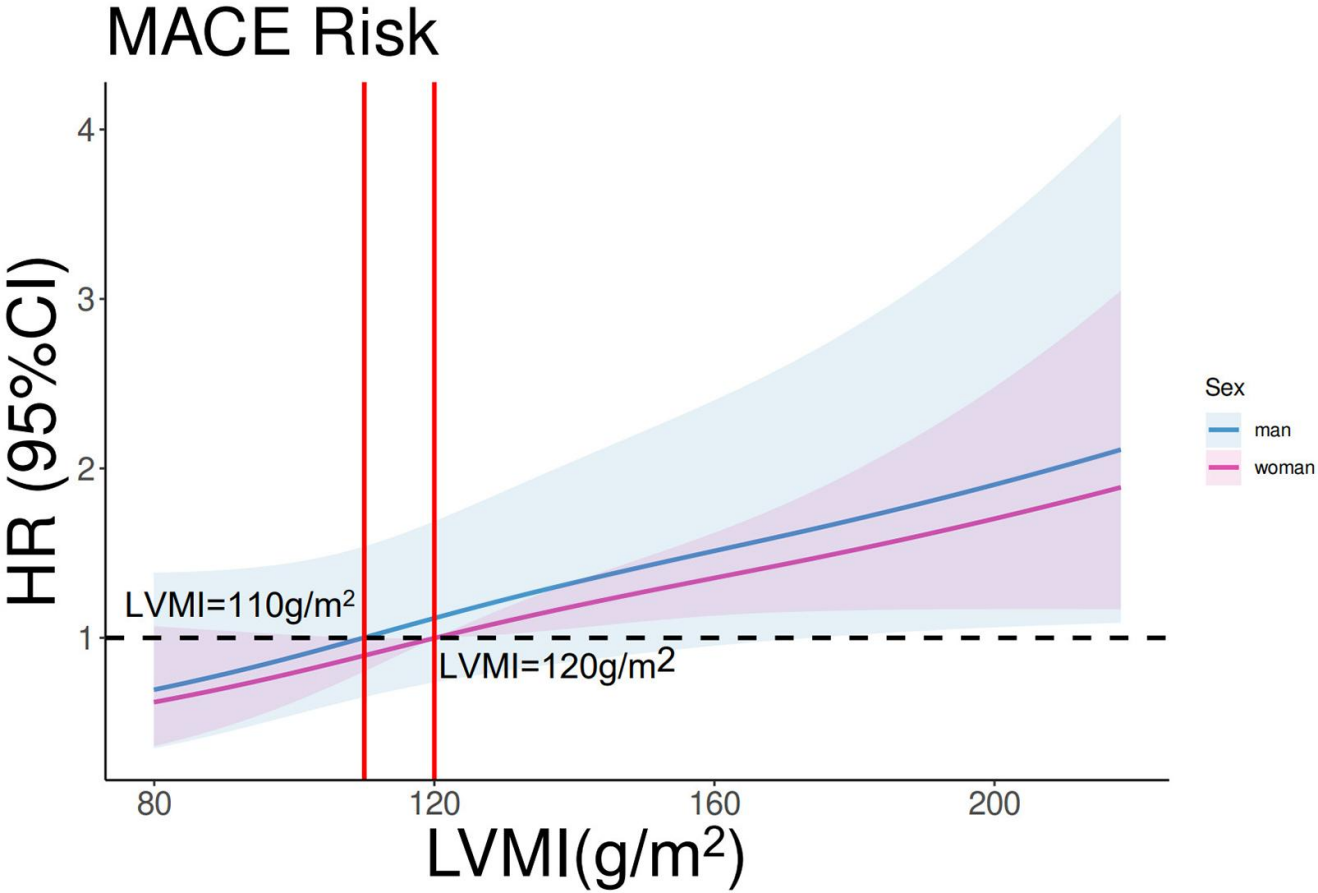
Restricted cubic spline analysis on the association between LVMI and MACE. *p* for linearity = 0.48. The purple line and shades represent the HR and CI for MACE risk in women. The blue line and shades represent the HR and CI for MACE risk in men. The left vertical red line equals LVMI = 110 g/m^2^, and the right vertical red line equals LVMI = 120 g/m^2^.

**Figure 3:**
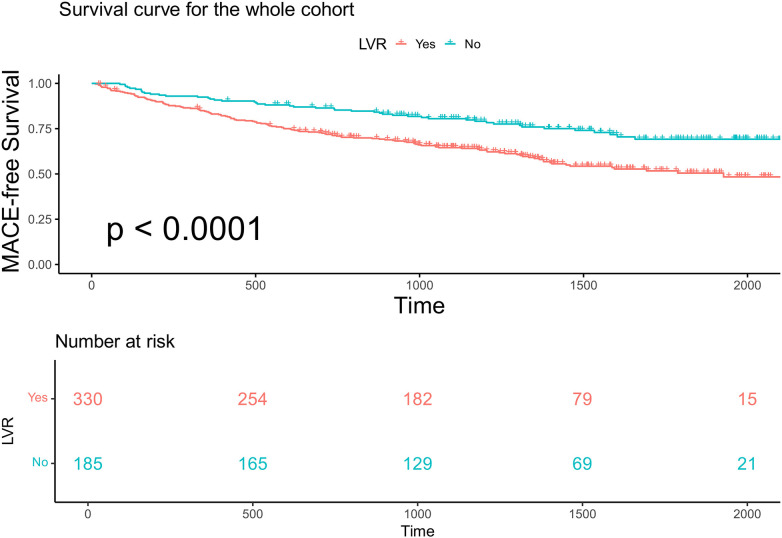
Kaplan–Meier analysis and the risk table according to whether patients have advanced LVR or not in the whole cohort. Log rank *p* < 0.0001. The green line shows patients with advanced LVR. Of 330 patients, 254, 182, 70, and 15 were still free from MACE and were tracked at 500 days, 1,000 days, 1,500 days, and 2,000 days, respectively. The red line shows patients without advanced LVR. Of 185 patients, 165, 129, 69, and 21 were still free from MACE and were tracked at 500 days, 1,000 days, 1,500 days, and 2,000 days, respectively.

In the entire cohort, continuous LVMI was a significant predictor of MACE in the entire cohort after adjustment for sex, age, LVEF, and NT-proBNP [multivariable Cox regression, *p* < 0.001, HR 1.007 per unit increase, 95% confidence interval (CI) 1.003–1.0116]. Restricted cubic spline analysis (three knots) revealed a linear increase in the HR for MACE with increasing LVMI values in both male and female patients (*p* for linearity = 0.48; Figure 2). Based on this analysis, sex-specific LVMI thresholds were identified: 110 g/m^²^ for men and 120 g/m^²^ for women. A total of 330 patients met these criteria for advanced LVR. Patients with advanced LVR had a significantly higher risk of MACE than those without advanced LVR (log-rank *p* < 0.01; Figure 3). Baseline characteristics of these patients are summarized in Table 1. Compared to patients without advanced LVR, those with advanced LVR were more likely to be male, have a non-ischemic etiology, lower LVEF, lower eGFR, and more impaired myocardial work indices.

**Table 1:** Differences in key baseline and myocardial work variables in according to LVMI.

Variables	Category	LVR (*n* = 330)	Non-LVR (*n* = 185)	*p*
Age, years	Mean (SD)	60.8 (12.3)	61.3 (10.8)	0.668
Female, no. (%)		35 (10.6)	35 (18.9)	0.012
BMI, kg/m^2^	Mean (SD)	24.9 (3.6)	25.1 (3.7)	0.447
NYHA class	1	3 (0.9)	3 (1.6)	<0.001
	2	118 (35.8)	112 (60.5)	
	3	175 (53.0)	65 (35.1)	
	4	34 (10.3)	5 (2.7)	
LVEF, %	Mean (SD)	35.9 (8.6)	44.1 (5.5)	<0.001
LVMI, g/m^2^	Mean (SD)	144.5 (30.8)	97.7 (10.4)	<0.001
HF etiology	Ischemic	169 (51.2)	149 (80.5)	<0.001
	Non-ischemic	161 (48.8)	36 (19.5)	
Diabetes, no. (%)		126 (38.2)	80 (43.2)	0.303
eGFR, mL/min/1.73 m^²^	Mean (SD)	80.0 (27.9)	86.3 (25.7)	0.013
NT-proBNP, pg/mL	Mean (SD)	3,467.0 (5,284.3)	2,640.0 (4,527.9)	0.074
RAAS inhibitors use, no. (%)		270 (81.8)	142 (76.8)	0.207
Beta blocker use, no. (%)		292 (88.5)	165 (89.2)	0.922
GWI, mmHg%	Mean (SD)	862.1 (455.3)	1,087.1 (406.0)	<0.001
GWE, %	Mean (SD)	0.79 (0.11)	0.84 (0.09)	<0.001
GCW, mmHg%	Mean (SD)	1,046.7 (493.2)	1,265.5 (446.8)	<0.001
GLS, %	Mean (SD)	9.5 (3.4)	11.9 (3.3)	<0.001

LVR, left ventricular remodeling; SD, standard deviation; BMI, body mass index; NYHA, New York Heart Association; LVEF, left ventricular ejection fraction; LVMI, left ventricular mass index; HF, heart failure; eGFR, estimated glomerular filtered rate; NT-proBNP, N-terminal pro-B-type natriuretic peptide; RAAS, renin–angiotensin–aldosterone system; GWI, global work index; GWE, global work efficiency; GCW, global constructive work; GLS, global longitudinal strain.

### 3.3 Obesity paradox in advanced LVR

**Figure 4:**
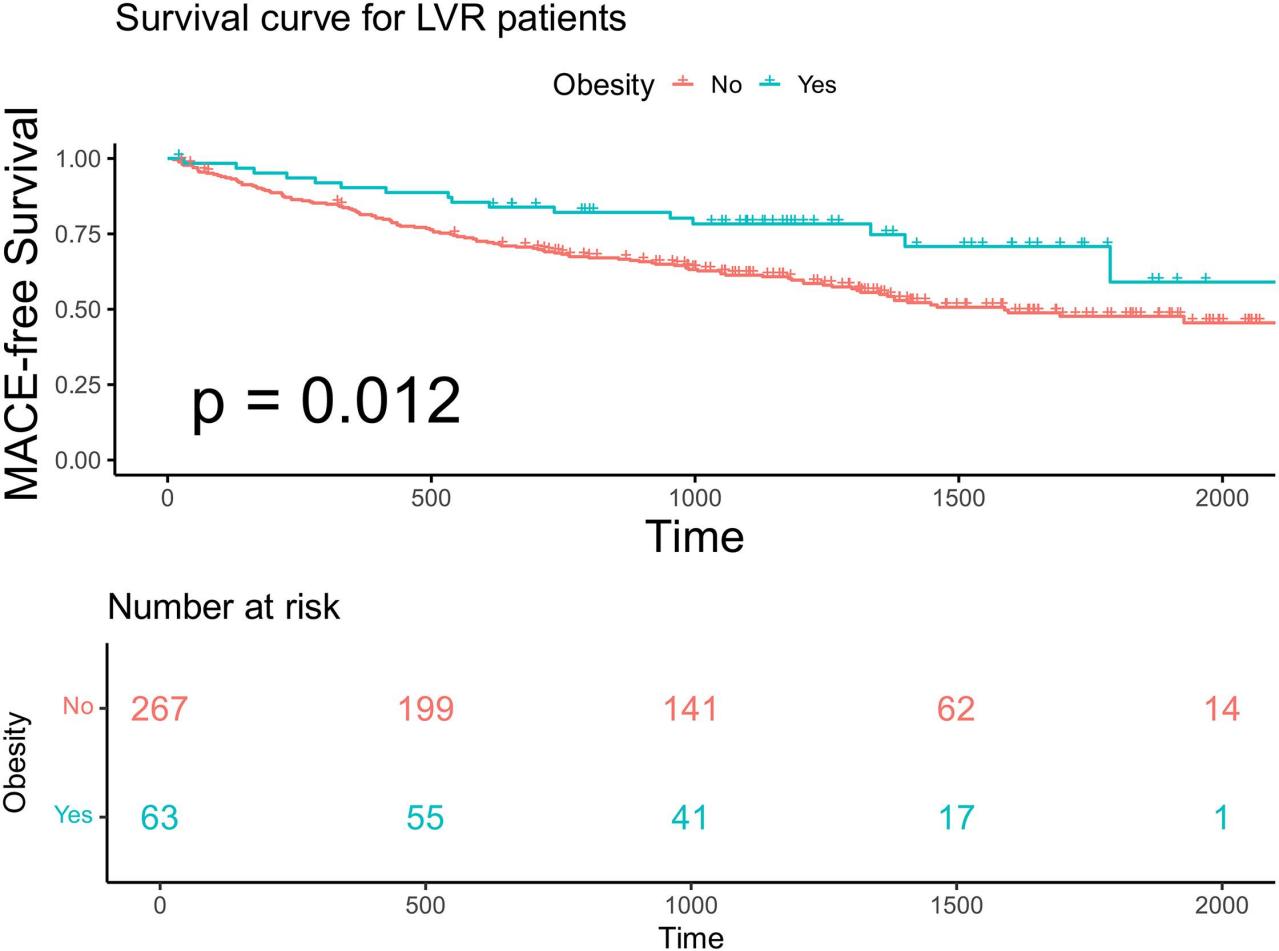
Kaplan–Meier analysis and the risk table according to BMI in patients with advanced LVR. Log rank *p* = 0.012. The green line shows patients with obesity. Of 63 patients, 55, 41, 17, and 1 were still free from MACE and were tracked at 500 days, 1,000 days, 1,500 days, and 2,000 days, respectively. The red line shows patients without obesity. Of 267 patients, 199, 141, 62, and 14 were still free from MACE and were tracked at 500 days, 1,000 days, 1,500 days, and 2,000 days, respectively.

We specifically analyzed the obesity paradox in 330 patients with advanced LVR. Kaplan–Meier analysis showed that obese patients (BMI ≥ 28 kg/m^²^) had a significantly higher MACE-free survival compared with non-obese patients (log-rank *p* = 0.012; Figure 4).

### 3.4 Predictive value of GWI

**Figure 5:**
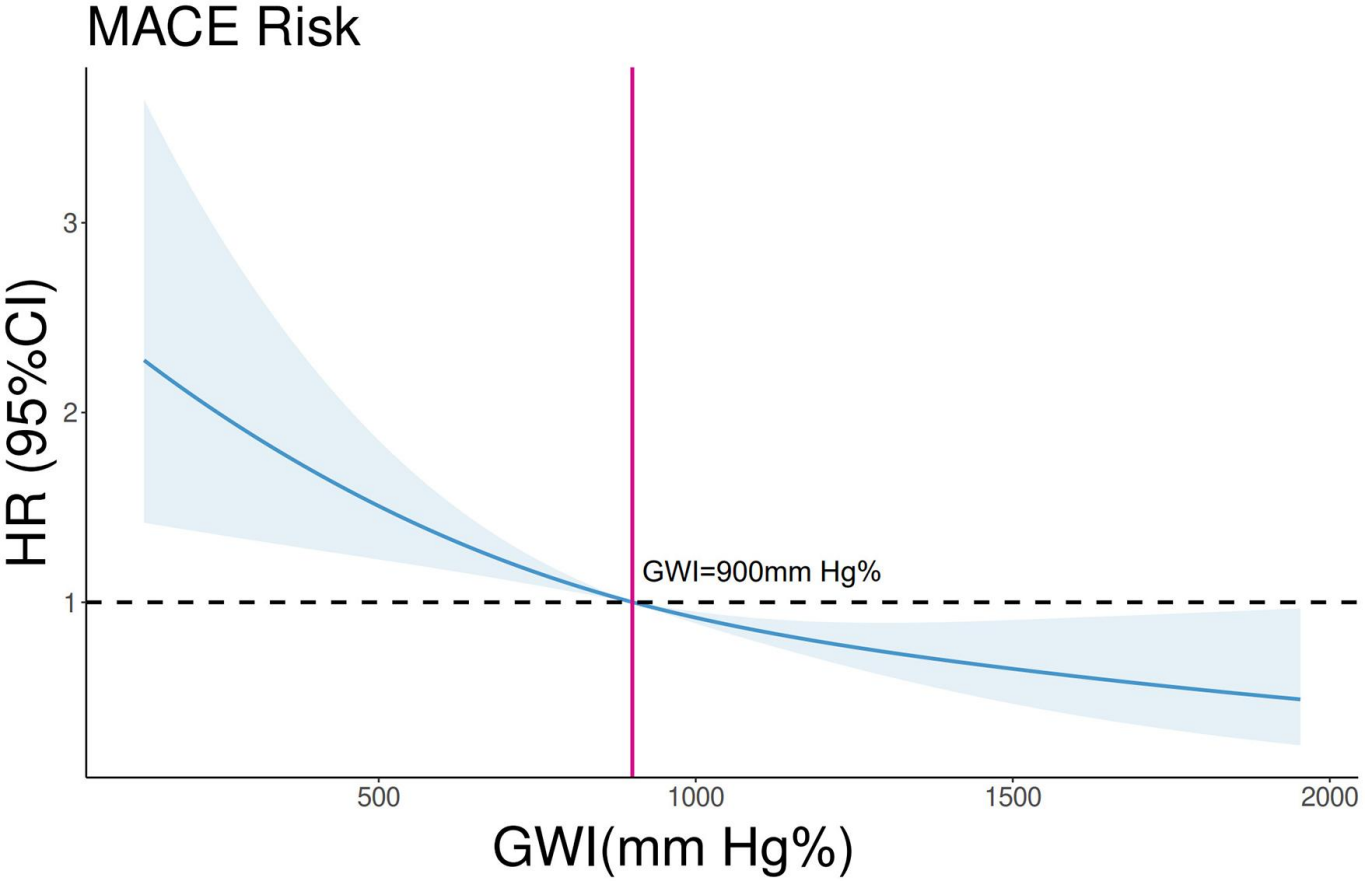
Restricted cubic spline analysis on the association between GWI and MACE in the whole cohort. *p* for linearity = 0.48. The blue line and shades represent the HR and CI for MACE risks, respectively. The vertical red line equals GWI = 900 mmHg%.

**Figure 6:**
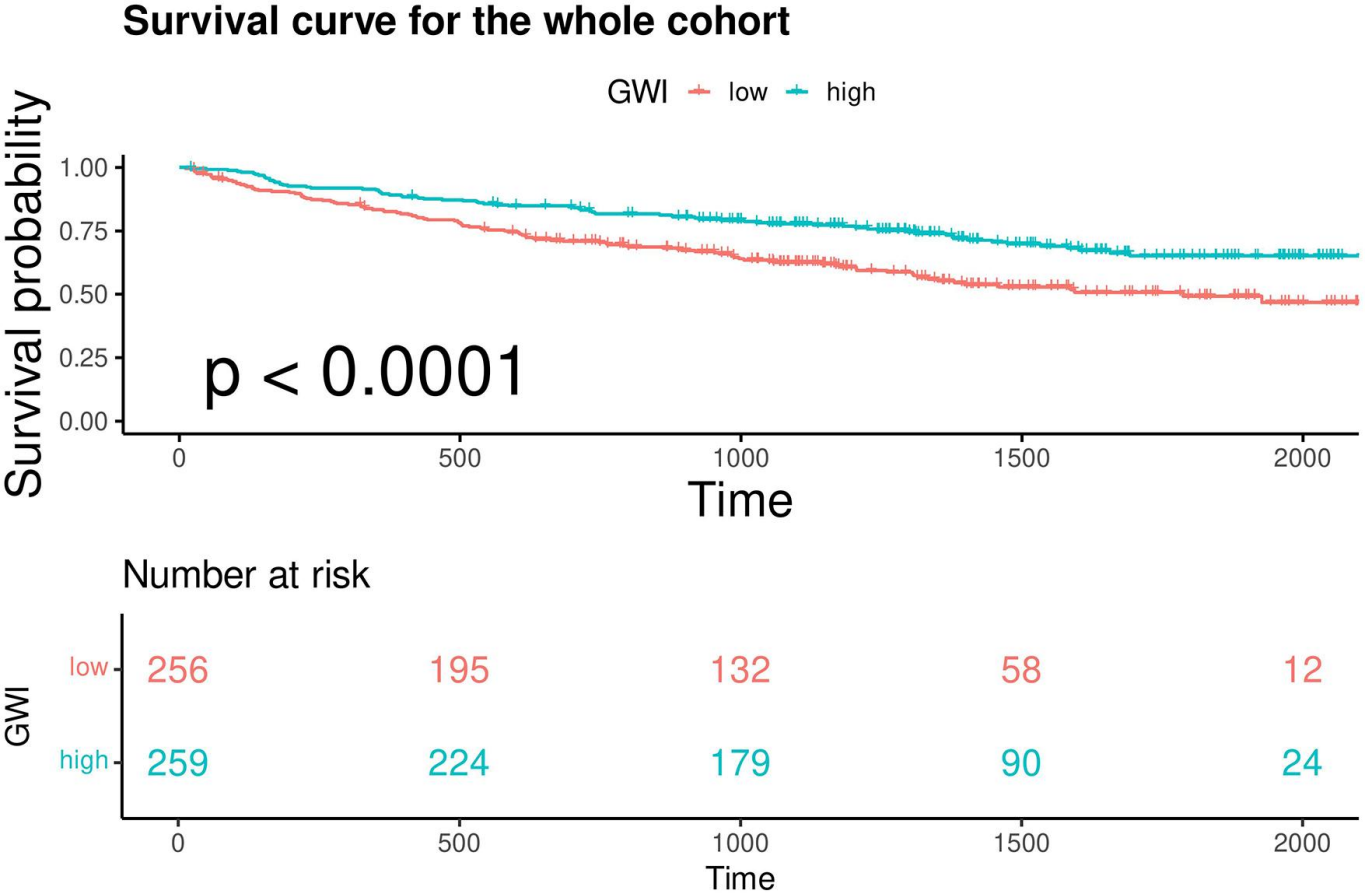
Kaplan–Meier analysis and the risk table of patients with GWI ≥ 900 mmHg% (high) and GWI < 900 mmHg% (low) in the whole cohort. Log rank *p* < 0.0001. The green line shows patients with GWI ≥ 900 mmHg%. Of 259 patients, 224, 179, 90, and 24 were still free from MACE and were tracked at 500 days, 1,000 days, 1,500 days, and 2,000 days, respectively. The red line shows patients with GWI < 900 mmHg%. Of 256 patients, 195, 132, 58, and 12 were still free from MACE and were tracked at 500 days, 1,000 days, 1,500 days, and 2,000 days, respectively.

In the overall cohort, continuous GWI was also a predictor of MACE (univariable Cox regression, HR 0.999 per unit increase, 95% CI 0.9988–0.9994, *p* < 0.001). A linear inverse relationship between GWI and the HR for MACE was confirmed by restricted cubic spline analysis (three knots, *p* for linearity = 0.48; Figure 5). A GWI value above 900 mmHg% was associated with better myocardial work capacity and a lower risk of MACE (log-rank *p* < 0.001; Figure 6). This association was confirmed by Cox regression analysis (GWI ≥ 900 mmHg% vs. GWI < 900 mmHg%: univariable HR 0.56, 95% CI 0.416–0.752, *p* < 0.001; multivariable model 2 HR 0.58, 95% CI 0.420–0.794, *p* < 0.001; Table 2). Based on this threshold, patients with advanced LVR were stratified into higher GWI (GWI ≥ 900 mmHg%, *n* = 139) and lower GWI (GWI < 900 mmHg%, *n* = 191) subgroups. Their comparative characteristics are summarized in Table 3. Patients in the higher GWI group were more likely to have an ischemic etiology, higher LVEF, lower New York Heart Association class, lower NT-proBNP levels, more favorable LV geometry, and better myocardial work indices. The protective prognostic effect of higher GWI remained significant in patients with advanced LVR (multivariable model 2 HR 0.54, 95% CI 0.370–0.799, *p* < 0.001, Table 2).

**Table 2:** Cox proportional hazards models for the relationship between GWI and MACE.

Variables	Univariate Cox regression HR (CI)	*p* value	Multivariable Cox regression model 1 HR (CI)	*p* value	Multivariable Cox regression model 2 HR (CI)	*p* value
Whole cohort						
GWI (continuous)	0.9991 (0.9988–0.9995)	<0.001	0.9991 (0.9988–0.9994)	<0.001	0.9992 (0.9988–0.9996)	<0.001
GWI > 900 mmHg%	0.56 (0.416–0.752)	<0.001	0.564 (0.4194–0.758)	<0.001	0.5773 (0.4199–0.7935)	<0.001
Advanced LVR						
GWI (continuous)	0.9993 (0.9989–0.9997)	<0.001	0.9993 (0.9989–0.9997)	<0.001	0.9993 (0.9989–0.9998)	0.002
GWI > 900 mmHg%	0.5596 (0.3912–0.8004)	0.001	0.5658 (0.3952–0.8101)	0.002	0.5443 (0.3695–0.7992)	<0.001

Model 1: adjusted for age and sex. Model 2: adjusted for hypertension, diabetes mellitus, ischemic etiology, estimated glomerular filtration rate, renin–angiotensin–aldosterone system, beta-blocker and mineralocorticoid receptor antagonist medication use, and N-terminal pro-brain natriuretic peptide. HR, hazard ratio; CI, confidential interval; BMI, body mass index; GWI, global work index; LVR, left ventricular remodeling.

**Table 3:** Differences in key baseline and myocardial work variables in GWI subgroups.

Variables	Category	GWI < 900 mmHg% (*n* = 191)	GWI ≥ 900 mmHg% (*n* = 139)	*p*
Age, years	Mean (SD)	60.9 (12.6)	60.8 (11.9)	0.958
Female, no. (%)		22 (11.5)	13 (9.4)	0.653
BMI, kg/m^2^	Mean (SD)	24.9 (3.8)	24.9 (3.3)	0.945
NYHA class	1	2 (1.0)	1 (0.7)	0.002
	2	55 (28.8)	63 (45.3)	
	3	106 (55.5)	69 (49.6)	
	4	28 (14.7)	6 (4.3)	
LVEF, %	Mean (SD)	31.5 (7.4)	42.0 (5.9)	<0.001
LVMI, g/m^2^	Mean (SD)	148.2 (31.6)	139.4 (29.1)	0.010
HF etiology	Ischemic	84 (44.0)	85 (61.2)	0.003
	Non-ischemic	107 (56.0)	54 (38.8)	
Diabetes, no. (%)		75 (39.3)	51 (36.7)	0.718
eGFR, mL/min/1.73 m^²^	Mean (SD)	79.3 (28.2)	80.9 (27.7)	0.610
NT-proBNP, pg/mL	Mean (SD)	4,065.6 (6,012.3)	2,644.5 (3,953.0)	0.016
RAAS inhibitors use, no. (%)		162 (84.8)	108 (77.7)	0.131
Beta blocker use, no. (%)		172 (90.1)	120 (86.3)	0.384
GWI, mmHg%	Mean (SD)	543.4 (222.3)	1,300.1 (303.9)	<0.001
GWE, %	Mean (SD)	0.74 (0.11)	0.86 (0.06)	<0.001
GCW, mmHg%	Mean (SD)	709.5 (254.4)	1,510.2 (342.2)	<0.001
GLS, %	Mean (SD)	7.3 (2.1)	12.4 (2.5)	<0.001

GWI, global work index; SD, standard deviation; BMI, body mass index; NYHA, New York Heart Association; LVEF, left ventricular ejection fraction; LVMI, left ventricular mass index; HF, heart failure; eGFR, estimated glomerular filtered rate; NT-proBNP, N-terminal pro-B-type natriuretic peptide; RAAS, renin–angiotensin–aldosterone system; GWI, global work index; GWE, global work efficiency; GCW, global constructive work; GLS, global longitudinal strain.

### 3.5 Impact of obesity on prognosis in different GWI subgroups

**Figure 7:**
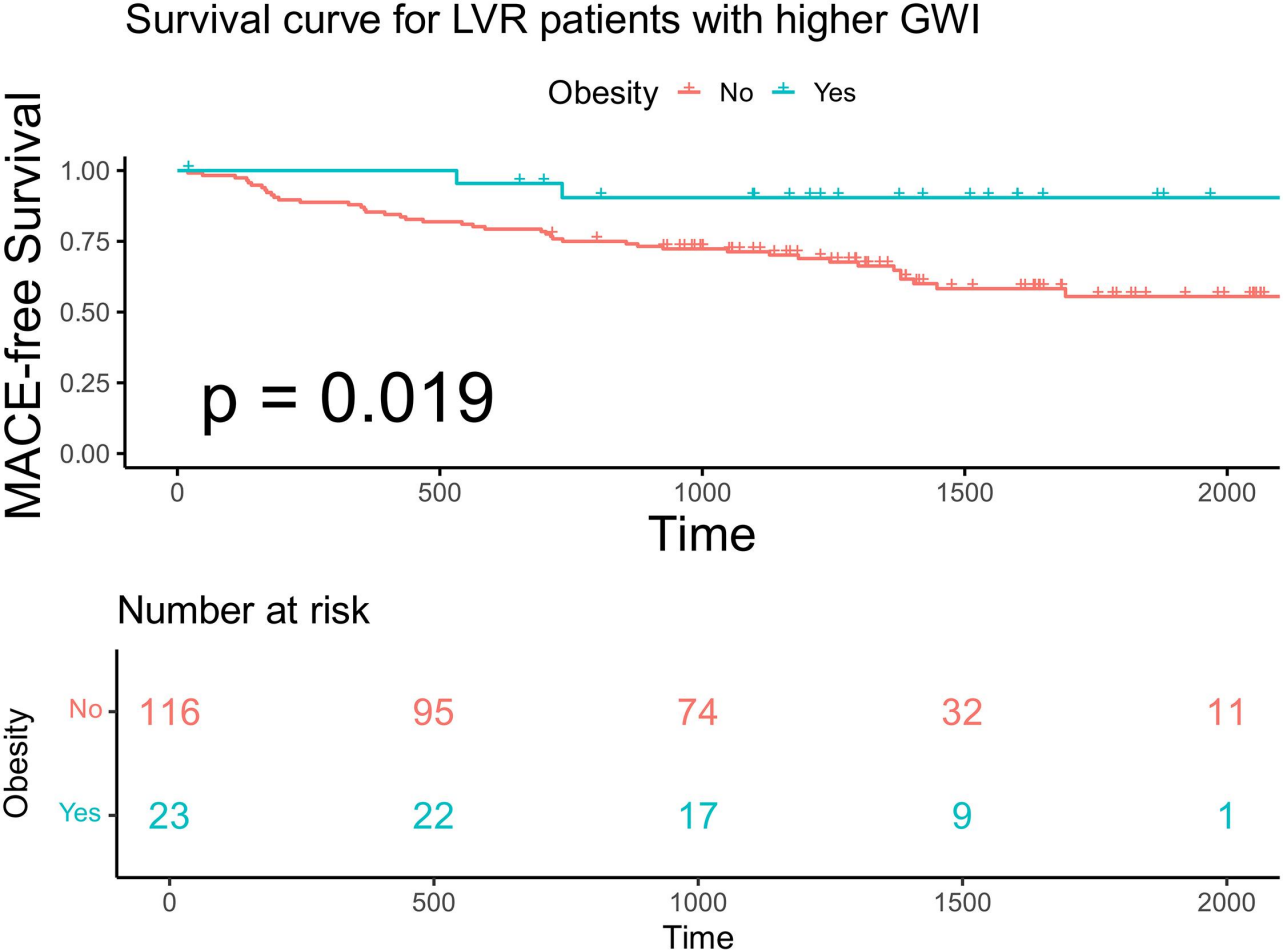
Kaplan–Meier analysis and the risk table according to BMI in advanced LVR patients with higher GWI. Log rank *p* = 0.019. The green line shows patients with obesity. Of 23 patients, 22, 17, 9, and 1 were still free from MACE and were tracked at 500 days, 1,000 days, 1,500 days, and 2,000 days, respectively. The red line shows patients without obesity. Of 116 patients, 95, 74, 32, and 11 were still free from MACE and were tracked at 500 days, 1,000 days, 1,500 days and 2,000 days, respectively.

**Figure 8:**
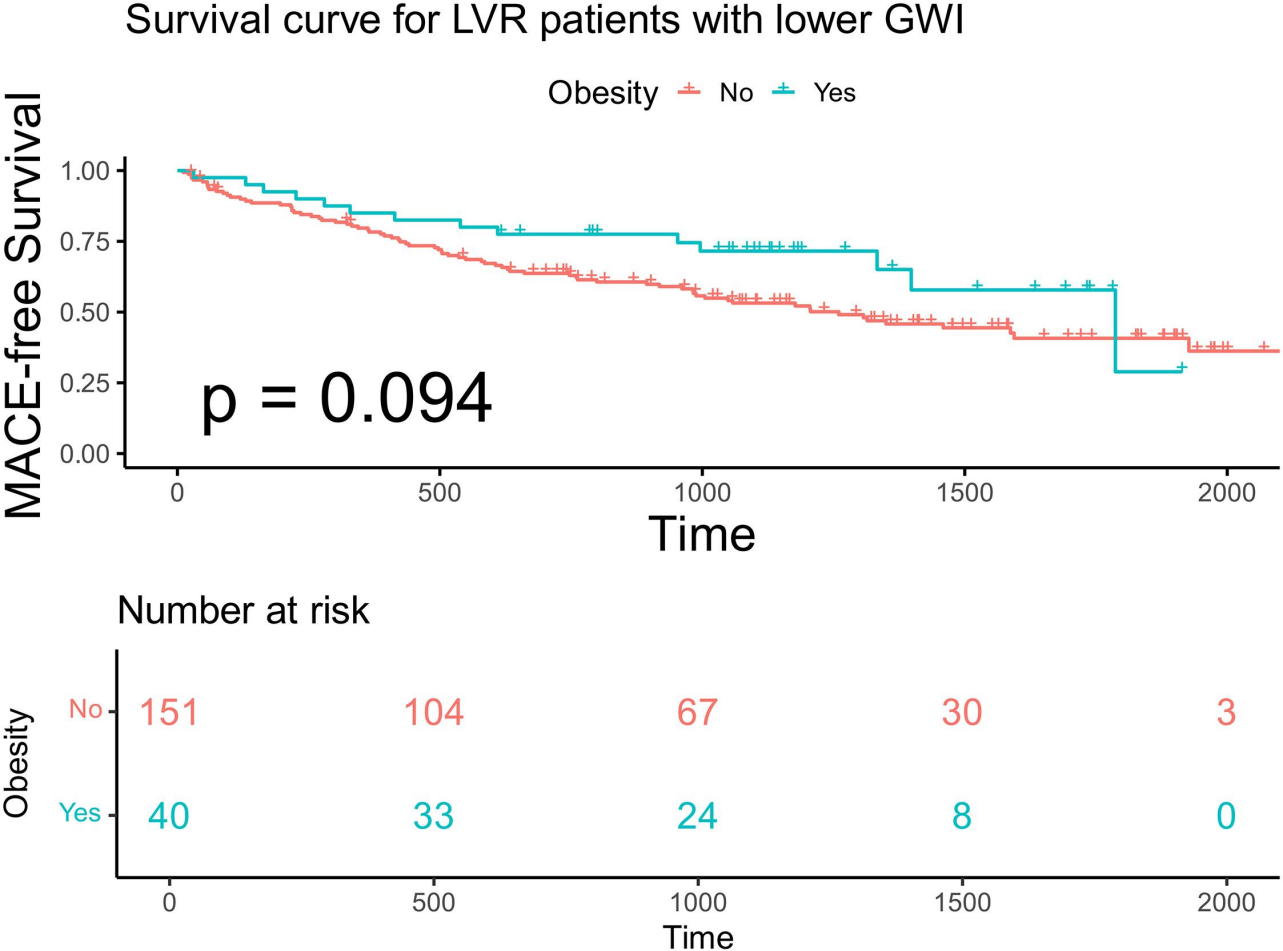
Kaplan–Meier analysis and the risk table according to BMI in advanced LVR patients with lower GWI. Log rank *p* = 0.019. The green line shows patients with obesity. Of 40 patients, 33, 24, 8, and 0 were still free from MACE and were tracked at 500 days, 1,000 days, 1,500 days, and 2,000 days, respectively. The red line shows patients without obesity. Of 151 patients, 104, 67, 30, and 3 were still free from MACE and were tracked at 500 days, 1,000 days, 1,500 days, and 2,000 days, respectively.

**Figure 9:**
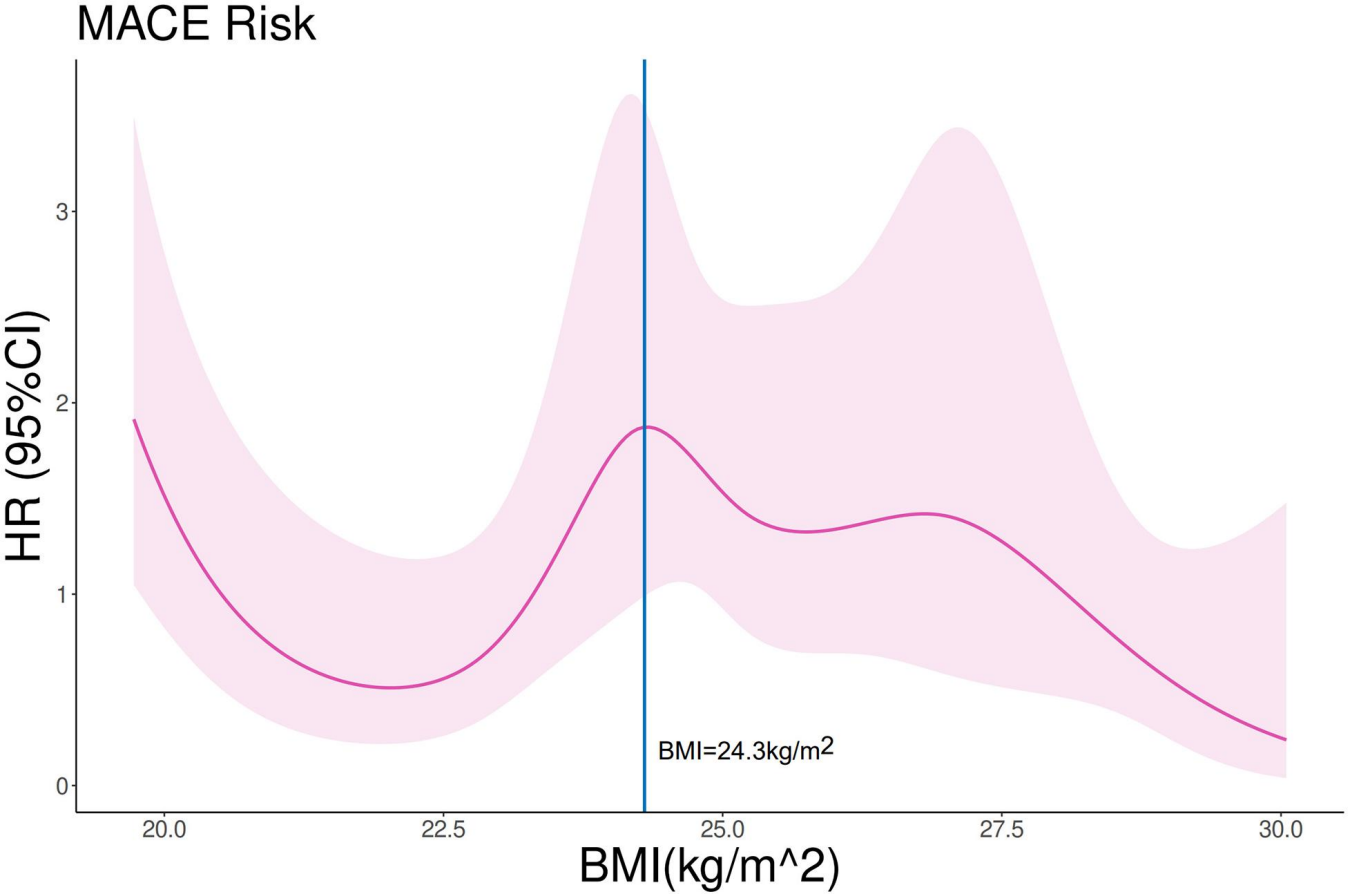
Restricted cubic spline analysis on the association between BMI and MACE in advanced LVR patients with higher GWI. *p* for linearity = 0.04. The purple line and shades represent the HR and CI for MACE risk, respectively. The vertical blue line equals BMI = 24.3 kg/m^2^.

Within each GWI subgroup, patients were further categorized according to obesity status (BMI ≥ 28 kg/m^²^). Baseline characteristics stratified by GWI and obesity status are presented in Table 4. Obese patients were younger than non-obese patients in both subgroups. In the higher GWI subgroup, obese patients demonstrated significantly better MACE-free survival than non-obese patients (*F* log-rank *p* = 0.019, Figure 7; multivariable model 2 HR 0.13; 95% CI 0.029–0.595, *p* = 0.008, Table 5). This survival advantage was not observed in the lower GWI subgroup (log-rank *p* = 0.094, Figure 8; multivariable model 2 HR 0.73, 95% CI 0.398–1.327, *p* = 0.299, Table 5). When analyzed as a continuous variable, BMI was a significant predictor of MACE in the higher GWI subgroup (univariable HR 0.894 per unit increase, 95% CI 0.810–0.987, *p* = 0.026). This association was attenuated after adjustment for age and sex (multivariable model 1 HR 0.899, 95% CI 0.807–1.001, *p* = 0.051) but remained significant after further adjustment for traditional cardiovascular risks (multivariable model 2 HR 0.889, 95% CI 0.807–0.980, *p* = 0.018). Restricted cubic spline analysis in the higher GWI group revealed a non-linear relationship between BMI and the HR (six knots, *p* for linearity = 0.04; Figure 9), with the highest risk observed at a BMI of 24.3 kg/m² and a progressive decline in HR with increasing BMI thereafter. In contrast, no significant association was found between continuous BMI and MACE in the lower GWI subgroup (univariable HR 0.982, 95% CI 0.932–1.036, *p* = 0.514; multivariable model 2 HR 0.998, 95% CI 0.940–1.059, *p* = 0.947).

**Table 4:** Differences in key baseline and myocardial work variables according to BMI classification in different GWI subgroup.

Variables	Category	Higher GWI			Lower GWI		
		BMI < 28 kg/m^2^	BMI ≥ 28 kg/m^2^	*p*	BMI < 28 kg/m^2^	BMI ≥ 28 kg/m^2^	*p*
Age, years	Mean (SD)	62.1 (11.2)	54.0 (13.3)	0.003	63.1 (11.4)	52.5 (13.6)	<0.001
Female, no. (%)		10 (8.6)	3 (13.0)	0.784	21 (13.9)	1 (2.5)	0.083
BMI, kg/m^2^	Mean (SD)	23.9 (2.4)	29.9 (2.2)	<0.001	23.4 (2.5)	30.4 (2.7)	<0.001
NYHA class	1	0 (0.0)	1 (4.3)	0.150	1 (0.7)	1 (2.5)	0.545
	2	54 (46.6)	9 (39.1)		41 (27.2)	14 (35.0)	
	3	57 (49.1)	12 (52.2)		86 (57.0)	20 (50.0)	
	4	5 (4.3)	1 (4.3)		23 (15.2)	5 (12.5)	
LVEF, %	Mean (SD)	42.1 (5.8)	41.5 (6.2)	0.620	31.4 (7.4)	31.9 (7.6)	0.658
LVMI, g/m^2^	Mean (SD)	138.5 (29.2)	143.9 (29.0)	0.421	149.3 (32.7)	144.3 (26.8)	0.376
HF etiology	Ischemic	76 (65.5)	9 (39.1)	0.033	69 (45.7)	15 (37.5)	0.454
	Non-ischemic	40 (34.5)	14 (60.9)		82 (54.3)	25 (62.5)	
Diabetes, no. (%)		41 (35.3)	10 (43.8)	0.615	59 (39.1)	16 (40.0)	1
eGFR, mL/min/1.73 m^²^	Mean (SD)	80.2 (27.4)	84.6 (29.5)	0.486	78.4 (28.6)	82.7 (26.8)	0.397
NT-proBNP, pg/mL	Mean (SD)	2,513.8 (3,798.5)	3,303.6 (4,695.9)	0.383	4,599.1 (6,575.7)	2,051.5 (2,130.0)	0.017
RAAS inhibitors use, no. (%)		91 (78.4)	17 (73.9)		124 (82.1)	38 (95.0)	0.077
Beta-blocker use, no. (%)		98 (84.5)	22 (95.7)		135 (89.4)	37 (92.5)	0.776
GWI, mmHg%	Mean (SD)	1,319.0 (311.8)	1,204.5 (243.7)	0.099	540.6 (231.6)	554.1 (185.2)	0.733
GWE, %	Mean (SD)	0.86 (0.06)	0.84 (0.06)	0.120	0.74 (0.11)	0.76 (0.09)	0.124
GCW, mmHg%	Mean (SD)	1,522.6 (358.6)	1,447.6 (240.1)	0.339	701.5 (264.2)	739.7 (213.7)	0.399
GLS, %	Mean (SD)	12.8 (2.5)	10.8 (2.1)	0.001	7.5 (2.2)	6.8 (1.9)	0.077

GWI, global work index; SD, standard deviation; BMI, body mass index; NYHA, New York Heart Association; LVEF, left ventricular ejection fraction; LVMI, left ventricular mass index; HF, heart failure; eGFR, estimated glomerular filtered rate; NT-proBNP, N-terminal pro-B-type natriuretic peptide; RAAS, renin–angiotensin–aldosterone system; GWI, global work index; GWE, global work efficiency; GCW, global constructive work; GLS, global longitudinal strain.

**Table 5:** Cox proportional hazard models for the relationship between BMI and MACE.

Variables	Univariate Cox regression HR (CI)	*p* value	Multivariable Cox regression model 1 HR (CI)	*p* value	Multivariable Cox regression model 2 HR (CI)	*p* value
Higher GWI group						
BMI (continuous)	0.894 (0.8104–0.9866)	0.026	0.899 (0.8072–1.0006)	0.051	0.8894 (0.8072–0.98)	0.018
BMI > 28 kg/m^2^	0.2137 (0.0518–0.8822)	0.033	0.2788 (0.0664–1.170)	0.081	0.1316 (0.0291–0.595)	0.008
Lower GWI group						
BMI (continuous)	0.982 (0.9316–1.0361)	0.514	1.002 (0.9432–1.064)	0.951	0.998 (0.9401–1.059)	0.947
BMI > 28 kg/m^2^	0.6169 (0.3487–1.092)	0.097	0.7374 (0.4055–1.341)	0.318	0.727 (0.3984–1.3268)	0.299

Model 1: adjusted for age and sex. Model 2: adjusted for hypertension, diabetes mellitus, ischemic etiology, estimated glomerular filtration rate, renin–angiotensin–aldosterone system, beta-blocker and mineralocorticoid receptor antagonist medication use, and N-terminal pro-brain natriuretic peptide. HR, hazard ratio; CI, confidential interval; BMI, body mass index; GWI, global work index.

## 4 Discussion

### 4.1 Key findings

This study yielded three principal findings, as summarized in the Graphical Abstract. First, we identified a distinct subgroup of patients with HF and advanced LVR using the echocardiographic variable LVMI. Second, we confirmed the presence of the obesity paradox within the high-risk subgroup. Third, we demonstrated that the myocardial work index GWI modulates the prognostic effect of BMI in patients with advanced LVR.

### 4.2 LVMI as a predictor for MACE

While normal LVMI values have been well-established in healthy individuals and patients with hypertension (7), the clinical significance of LVMI in HF populations remains less explored. LVMI is influenced by several factors, such as sex, body size, ethnicity, and physical activity level (13, 14). In our cohort, LVMI emerged as a strong independent predictor of MACE. We identified sex-specific prognostic thresholds: LVMI ≥ 110 g/m^²^ for men and ≥ 120 g/m^²^ for women. Patients meeting these criteria were classified as having advanced LVR for subsequent analyses. This subgroup exhibited more severe symptoms and poorer LV systolic dysfunction at baseline, validating the clinical relevance of this classification. The higher cutoff value identified for women is noteworthy. Baseline characteristics showed a balanced distribution of established risk factors (e.g., age, LVEF, and renal function) between women with LVMI ≥120 g/m^²^ and men with LVMI ≥110 g/m^²^ (Supplementary Table S1). This observation aligns with the typically better prognosis observed in women with heart failure with reduced ejection fraction in large clinical trials (15) and is consistent with our finding of a lower HR for MACE in women at any given LVMI level (Figure 2).

### 4.3 Obesity paradox in HF

BMI is the standard metric for defining obesity in clinical trials. For the Chinese adult population, a BMI ≥28 kg/m^²^ is recommended to define obesity, which is lower than the WHO criterion. This lower cutoff is justified by evidence indicating a higher body fat percentage and greater cardiovascular risk at equivalent BMI levels in Chinese adults compared with Western populations (16). As our study predominantly involved patients of Han Chinese ancestry, we employed the BMI ≥ 28 kg/m^²^ definition.

The obesity paradox in HF remains underexplored in Chinese populations. While a multinational Asian study noted the presence of the obesity paradox, Chinese participants accounted for only approximately 20% of its sample (17). Another study involving Chinese patients with HF reported the obesity paradox specifically in those with non-ischemic or dilated cardiomyopathy (18). Given the relationship between obesity and cardiac structure, we therefore focused on patients with advanced LVR. In this subgroup, dichotomization by obesity status revealed a survival advantage for obese patients. However, when BMI was analyzed as a continuous variable, it lost its independent prognostic value after adjustment for age and sex (multivariable model 1 HR 0.988, 95% CI 0.945–1.032, *p* = 0.578). This finding underscores the complex and non-linear relationship between BMI and HF prognosis reported previously (19). The obesity paradox could be influenced by unmeasured confounding factors, such as physical activity and cardiorespiratory fitness (20, 21), which are often better preserved in obese HF patients and may contribute to the observed survival advantage. This observation suggests that BMI alone has limited utility in fully explaining the obesity paradox.

### 4.4 GWI and its impact on the prognosis of obesity

The GWI, derived from non-invasive pressure-strain loop analysis, correlates well with established prognostic markers, such as NT-proBNP and peak oxygen consumption (22), and offers the advantage of incorporating afterload. In our overall cohort, higher GWI (GWI ≥ 900 mmHg%) was associated with a better prognosis. This threshold is higher than the 750 mmHg% reported by Wang et al. (23), a discrepancy attributed to differences in study populations (LVEF cutoff  ≤50% in our study vs. ≤40% in theirs) and the longer follow-up duration.

We further investigated the influence of GWI on the obesity paradox in patients with advanced LVR. The survival benefit of obesity was evident only in patients with preserved myocardial work (higher GWI), implying that the obesity paradox is conditional on adequate myocardial work capacity. Among patients with impaired GWI, the favorable effect of higher BMI was no longer evident.

A plausible explanation involves metabolic balance. GWI reflects cardiac metabolic demand, while obesity is associated with greater metabolic reserve (24). A favorable balance between demand and reserve may help sustain efficient cardiac performance. Furthermore, preserved GWI is linked to higher levels of physical activity (25). Obese patients with HF often retain greater muscle strength (26); thus, those with higher GWI may have a more favorable body composition (e.g., greater lean mass), contributing to better outcomes (27). Further research should explore whether personalized physical interventions, guided by cardiopulmonary testing or wearable technology, can optimize body composition, enhance cardiac functional capacity, and improve clinical outcomes.

### 4.5 Study limitations

Several limitations of this study should be acknowledged. First, the single-center design and moderate sample size limit the generalizability of our findings. Second, obesity was defined solely by BMI. Additional anthropometric measurements (e.g., waist circumference, body fat percentage) might provide further insights into the obesity paradox and could attenuate the observed associations (19). Third, our cohort was predominantly male. Given that the effect of obesity may differ by sex, this imbalance may have influenced the results (1). Fourth, BMI and GWI were assessed only at baseline; serial changes in these parameters, which may be related to prognosis, were not captured. Fifth, as this was a *post hoc* analysis, data on important confounders—such as physical activity levels and cardiopulmonary exercise testing parameters, which are known to modulate the obesity paradox—were not available (20). Future prospective studies should incorporate these assessments to strengthen the validity of conclusions and guide clinical management. Finally, our study included relatively few patients with severe obesity or underweight, limiting the applicability of our findings to these extremes.

## 5 Conclusion

In patients with HF and advanced LVR, the relationship between BMI and prognosis is modified by myocardial work capacity, as measured by the GWI. An obesity paradox was observed only in patients with preserved GWI (≥900 mmHg%), who exhibited a lower risk of adverse outcomes. In contrast, no survival benefit was associated with higher BMI in patients with impaired GWI. Therefore, the prognostic value of BMI in HF should be interpreted in the context of concurrent myocardial work evaluation to enable more accurate risk stratification.

## Data Availability

The raw data supporting the conclusions of this article will be made available by the authors, without undue reservation.
