# Supplementary material for: Impact of global work index on obesity paradox in heart failure with advanced left ventricle remodeling
Source: Front Cardiovasc Med. 2026 Feb 17;13:1693862. doi: 10.3389/fcvm.2026.1693862 (PMC12953510; doi:10.3389/fcvm.2026.1693862)
Supplement: Supplementary file 1 [file Table1.docx]

**Supplementary Table 1 - Differences in Key Baseline and Myocardial Work Variables in LVR patients According to Sex**

|  |  | Female(n=35) | Male(n=295) | p |
| --- | --- | --- | --- | --- |
| Age, years | Mean (SD) | 63.1 (13.2) | 60.6 (12.2) | 0.253 |
| BMI, kg/m^2^ | Mean (SD) | 22.6 (4.3) | 25.1 (3.4) | <0.001 |
| NYHA class | 1 | 0 (0.0) | 3 (1.0) | 0.068 |
|  | 2 | 6 (17.1) | 112 (38.0) |  |
|  | 3 | 23 (65.7) | 152 (51.5) |  |
|  | 4 | 6 (17.1) | 28 (9.5) |  |
| LVEF, % | Mean (SD) | 35.3 (8.4) | 36.0 (8.6) | 0.639 |
| HF etiology | Ischemic | 8 (22.9) | 161 (54.6) | 0.001 |
|  | Non-ischemic | 27 (77.1) | 134 (45.4) |  |
| Diabetes, No (%) |  | 12(34.3) | 114(38.6) | 0.751 |
| eGFR, mL/min/1.73 m² | Mean (SD) | 78.6 (35.3) | 80.2 (27.0) | 0.761 |
| NT-proBNP, pg/ml | Mean (SD) | 5092.3 (7019.9) | 3274.2 (5019.7) | 0.054 |
| RAAS inhibitors use, No (%) | | 27 (77.1) | 243 (82.4) | 0.598 |
| Beta blocker use, No (%) | | 31 (88.6) | 261 (88.5) | 1 |
| GWI, mm Hg% | Mean (SD) | 751.7 (461.7) | 875.2 (453.5) | 0.129 |
| GWE, % | Mean (SD) | 0.75 (0.13) | 0.79 (0.10) | 0.014 |
| GCW, mm Hg% | Mean (SD) | 944.5 (525.6) | 1058.9 (488.8) | 0.195 |
| GLS, % | Mean (SD) | 9.3 (3.7) | 9.5 (3.4) | 0.732 |
| LVR, left ventricular remodeling; SD, standard deviation; BMI, body mass index; NYHA, New York Heart Association; LVEF, left ventricular ejection fraction; LVMI, left ventricular mass index; HF, heart failure; eGFR, estimated glomerular filtered rates; NT-proBNP, N-terminal pro-B-type natriuretic peptide; RAAS, Renin-angiotensin-aldosterone system; GWI, global work index; GWE, global work efficiency; GCW, global constructive work; GLS, global longitudinal strain | | | | |
